# Dose and time-dependence of acute intermittent theta-burst stimulation on hippocampus-dependent memory in parkinsonian rats

**DOI:** 10.3389/fnins.2023.1124819

**Published:** 2023-02-14

**Authors:** Yixuan Wang, Jian Liu, Yanping Hui, Zhongheng Wu, Ling Wang, Xiang Wu, Yihua Bai, Qiaojun Zhang, Libo Li

**Affiliations:** ^1^Department of Rehabilitation Medicine, The Second Affiliated Hospital, Xi’an Jiaotong University, Xi’an, China; ^2^Department of Physiology and Pathophysiology, School of Basic Medical Sciences, Xi’an Jiaotong University Health Science Center, Xi’an, China

**Keywords:** intermittent theta-burst stimulation, Parkinson’s disease, hippocampus, memory, hippocampal theta rhythm

## Abstract

**Background:**

The treatment options for cognitive impairments in Parkinson’s disease (PD) are limited. Repetitive transcranial magnetic stimulation has been applied in various neurological diseases. However, the effect of intermittent theta-burst stimulation (iTBS) as a more developed repetitive transcranial magnetic stimulation paradigm on cognitive dysfunction in PD remains largely unclear.

**Objective:**

Our aim was to explore the effect of acute iTBS on hippocampus-dependent memory in PD and the mechanism underlying it.

**Methods:**

Different blocks of iTBS protocols were applied to unilateral 6-hydroxidopamine-induced parkinsonian rats followed by the behavioral, electrophysiological and immunohistochemical analyses. The object-place recognition and hole-board test were used to assess hippocampus-dependent memory.

**Results:**

Sham-iTBS and 1 block-iTBS (300 stimuli) didn’t alter hippocampus-dependent memory, hippocampal theta rhythm and the density of c-Fos- and parvalbumin-positive neurons in the hippocampus and medial septum. 3 block-iTBS (900 stimuli) alleviated 6-hydroxidopamine-induced memory impairments, and increased the density of hippocampal c-Fos-positive neurons at 80 min post-stimulation but not 30 min compared to sham-iTBS. Interestingly, 3 block-iTBS first decreased and then increased normalized theta power during a period of 2 h following stimulation. Moreover, 3 block-iTBS decreased the density of parvalbumin-positive neurons in the medial septum at 30 min post-stimulation compared to sham-iTBS.

**Conclusion:**

The results indicate that multiple blocks of iTBS elicit dose and time-dependent effects on hippocampus-dependent memory in PD, which may be attributed to changes in c-Fos expression and the power of theta rhythm in the hippocampus.

## 1. Introduction

Parkinson’s disease (PD) is a neurodegenerative disorder characterized by nigrostriatal dopaminergic deficiency. Apart from motor symptoms, PD patients suffer from a spectrum of additional symptoms including depression, anxiety and cognitive impairments. Cognitive impairment is one of the most prevalent non-motor symptoms and occurs at any stage of the disease, and can severely affect the quality of life of PD patients. A wide range of cognitive domains are affected in PD, including executive function, visuospatial abilities, attention, and memory (Aarsland et al., 2017). Although acetylcholinesterase inhibitor rivastigmine may play a positive role, evidence for medications to prevent or delay cognitive decline in PD is lacking (Sun and Armstrong, 2021). Thus, it is meaningful to develop novel therapeutic approaches for the treatment of cognitive impairment in PD.

Repetitive transcranial magnetic stimulation (rTMS) has been proposed to be a safe, painless and non-invasive therapy that alters neuronal activity in the cerebral cortex through rapidly changing magnetic fields. In the last two decades, the application of rTMS has been observed in a wide range of neurological and psychiatric diseases. rTMS may exert positive effects on cognitive function in Alzheimer’s disease and mild cognitive impairment (Nardone et al., 2014; Chou et al., 2020). A more developed rTMS paradigm, intermittent theta-burst stimulation (iTBS), during which stimulation was applied as 3 pulses at a frequency of 50 Hz-burst, repeated at 5 Hz, in a 2-s on/8-s off pattern, has been shown to induce a facilitatory effect on cortical excitability with lower intensity and shorter duration than regular high-frequency rTMS paradigm (Huang et al., 2005). So far, the results of clinical studies on the effects of iTBS on cognitive function in PD are inconsistent. Studies showed that single iTBS block didn’t affect working memory and executive function of PD patients (Hill et al., 2020), six sessions of iTBS over the dorsolateral prefrontal cortex tended to improve executive function in subjects with PD-mild cognitive impairment (Lang et al., 2020), and 10 sessions of iTBS over the right dorsolateral prefrontal cortex were able to promote metaphor comprehension in a right-handed PD patient with 9-year history (Tremblay et al., 2016). Therefore, the efficacy of multiple blocks of iTBS on cognitive function in PD is worth being explored. Considering the complex results obtained from previous studies on the role of iTBS in PD-related cognitive impairment, the underlying mechanism of these effects also deserve intensive investigation.

The hippocampus is an important brain region implicated in the pathogenesis of cognitive impairment in PD (Calabresi et al., 2013; Kouli et al., 2020; Becker et al., 2021; Jiang et al., 2021). Many studies have shown that regular rTMS or iTBS can improve cognition while altering hippocampal activity in various neurological diseases (Guo et al., 2017; Ma et al., 2019; Cai et al., 2020). For example, high frequency of rTMS was found to ameliorate cognitive impairment *via* enhancing hippocampal synaptic structural plasticity in naturally aged mice (Ma et al., 2019). Guo et al. (2017) applied rTMS in rats with ischemic stroke, they found that rTMS recovered cognitive impairment by suppressing apoptosis and enhancing neurogenesis in the hippocampus. With regard to iTBS, it was shown that iTBS enhanced cognitive function while changing the proliferation of astrocytes and microglia in the hippocampus in rats with cerebral small vessel disease (Cai et al., 2020). These studies indicate that regular rTMS or iTBS can enhance cognitive behavior *via* stimulation of the hippocampus. However, it still remains unclear whether iTBS can regulate cognitive behavior in PD by modulating hippocampal activity.

Thus, in the present study, we aimed to explore the effects of different blocks of iTBS on hippocampus-dependent memory in unilateral 6-hydroxidopamine (6-OHDA)-induced parkinsonian rats and the mechanism underlying it.

## 2. Materials and methods

### 2.1. Animals and drugs

Adult Male Sprague-Dawley rats (Experimental Animal Center of Xi’an Jiaotong University, Xi’an, China) weighing 270–320 g were used in this study (*n* = 130 rats). Room temperature was kept constant at 22 ± 2°C and the light/dark cycle was 12 h/12 h. Rats had free access to water and food. All experimental procedures were conducted according to the National Institute of Health Guide for the Care and Use of Laboratory Animals (NIH Publications No. 8,023, revised 1978), and approved by the Ethics Committee for Animal Experimentation of Xi’an Jiaotong University. All efforts were made to minimize the number of animals used and their suffering.

Desipramine hydrochloride, 6-OHDA hydrochloride, and apomorphine hydrochloride were purchased from Sigma–Aldrich (Sigma–Aldrich, St. Louis, MO, USA). 6-OHDA was dissolved in artificial cerebrospinal fluid containing 0.02% ascorbic acid; apomorphine was prepared in saline with 0.02% ascorbic acid; desipramine was dissolved in saline. These drugs were prepared on the day of use.

### 2.2. Experimental procedures

**FIGURE 1:**
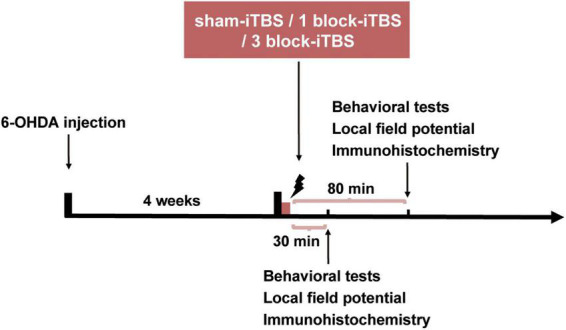
The timeline of the experiments. Four weeks after 6-OHDA injection, the parkinsonian rats were randomly allocated into three groups receiving sham-iTBS, 1 block-iTBS (one iTBS block) or 3 block-iTBS (three blocks of iTBS), respectively, all three groups were subjected to behavioral tests, LFP recordings, histology and immunohistochemistry at 30 or 80 min post-stimulation.

Figure 1 shows the timeline of the experiments. Four weeks after 6-OHDA injection, the lesioned rats which passed the apomorphine-induced contralateral rotation test were divided into three groups according to the number of pulses received: sham-iTBS, 1 block-iTBS (one iTBS block) and 3 block-iTBS (three blocks of iTBS). All three groups were subjected to behavioral tests, histology and immunohistochemistry at 30 or 80 min post-stimulation. In the specific case of behavioral tests including the object-place recognition (OPR) and hole-board (HB) test, the first trial of the two tests started at 30 or 80 min after real (different blocks) or sham-iTBS. Besides, hippocampal local field potential (LFP) was recorded in all three groups for 3 h, including 1 h pre-stimulation baseline and 2 h post-stimulation recordings 4 weeks after 6-OHDA injection.

### 2.3. Unilateral 6-OHDA lesions in the MFB

The unilateral 6-OHDA lesions of the MFB in rats were carried out as previously described (Carvalho et al., 2013). Rats were anesthetized with sodium pentobarbital (40 mg/kg, i.p.) followed by the injection of desipramine (25 mg/kg, i.p.) to protect noradrenergic neurons. Then the rats were placed on a stereotaxic frame (SN-2N; Narishige, Tokyo, Japan) and 6-OHDA (12 μg/4 μl) was injected into the left MFB (AP –4.4 mm, ML –1.2 mm, DV –7.8 mm; Paxinos and Watson, 1998). After the injection, the glass pipette was left in place for 5 min to allow diffusion. Two weeks later, rats were subcutaneously injected with apomorphine (0.05 mg/kg, s.c.) to verify the effectiveness of 6-OHDA lesion (Wang et al., 2009) and those showing more than 20 contralateral turns per 5 min were selected for further experiments. All rats used in the following experiments turned consistently toward right of >30 turns per 5 min.

### 2.4. iTBS

Four weeks after 6-OHDA injection, the lesioned rats were randomized into following groups (*n* = 21-22 rats/group): 30 min post sham-iTBS, 80 min post sham-iTBS, 30 min post 1 block-iTBS, 80 min post 1 block-iTBS, 30 min post 3 block-iTBS, and 80 min post 3 block-iTBS, and then received different iTBS sessions in waking relaxation state after a familiarization session. Briefly, for familiarization, the rats were gently handled, placed in a custom-made restraint device with head fixed and eyes covered, and trained to adapt to artifact noise and scalp sensation induced by iTBS daily for 3 days. During the stimulation period, different blocks of iTBS were applied using a Rui Chi magnetic stimulator (CCY–IA, YIRD, Wuhan, China) and a circular coil (inner diameter, 2.5 cm; outer diameter, 6.4 cm; YIRD, Wuhan, China) specific for rats. The coil was held parallel to the cranium with the center located 5 mm above the intersection of the sagittal suture and interaural line (Labedi et al., 2014). 1 block-iTBS consisted of 10 trains of 10 bursts (3 pulses, 50 Hz) repeated at 5 Hz with a train interval of 8 s (300 pulses; Huang et al., 2005). 3 block-iTBS were applied at 15-min intervals between each block (900 pulses). Stimulus intensity was set to 30% of maximal stimulator output. In this procedure, the stimulation conditions were almost identical, and the stimulation intensity was about 80% of motor threshold. For sham stimulation, we tilted the coil perpendicular (90°rotation) to the cranium and located the coil edge 20 cm apart from the scalp.

### 2.5. Behavioral tests

The behavioral tests were conducted 4 weeks after 6-OHDA injection. All the tests were performed in an isolated room between 9:00 and 11:00 a.m., and recorded using a digital camera (HR–550E, Sony, Tokyo, Japan).

#### 2.5.1. OPR task

The OPR task is utilized to assess the detection of novel spatial position of a known object and is critically dependent on the hippocampus (Sawangjit et al., 2018). The apparatus (40 × 40 × 40 cm) was an open box made of opaque gray Plexiglas. There were visual cues on the walls of the testing room in the visual field of the rats. The objects for exploration were two identical glass jars heavy enough not to be moved by the rat. The rats were handled for 10 min daily for 3 days to minimize the disruption of stress and novelty. Then the rats were successively allowed to explore for 10 min in the empty apparatus without objects for habituation. On the day after habituation, the experiment started with the encoding phase of the OPR task. The rats were allowed to explore two identical objects positioned in the back left and right corners of the apparatus for 10 min. For testing retrieval on the OPR task, 2 h later, one object remained in the same place as in the encoding phase, and the other was moved to a novel location. During the testing phase, the rats had 5 min to explore the apparatus. After each phase, the apparatus and objects were wiped with 20% alcohol. The rat was scored as “interacting” with the object when its nose was in contact with the object or directed toward the object within 2 cm. During the retrieval test, the time a rat spent “interacting” with each object was measured, both manually and using ANY-maze tracking software (Stoelting Co., Wood Dale, IL, USA), and converted into a discrimination ratio which was calculated with the following formula: (time spent at displaced object – time spent at stationary object)/(time spent at displaced object + time spent at stationary object). A higher discrimination ratio reflects a better performance of OPR memory. For evaluating the dose and time dependent effects of iTBS on OPR memory, the encoding phase of the OPR task started 30 or 80 min after real or sham-iTBS (*n* = 7 rats/group).

#### 2.5.2. HB test

The HB test has been shown to involve the dorsal hippocampus and utilized to test the ability of rats to recognize environmental spatial novelty (Lemon and Manahan-Vaughan, 2006; Costa et al., 2012). The recording chamber measured 40 × 40 × 40 cm was made of opaque gray Plexiglas. A hole-board (39.8 × 39.8 × 5 cm) was inserted into the floor of the recording chamber before the test. Each corner of the hole-board contained a hole, 5.5 cm in diameter and 5 cm deep. On the other side, there were no holes. Dim illumination was provided by a white bulb (120 Lx) suspended 1.2 m above the apparatus. On the day before the first trial, the home cages were transferred to experiment room till the end and rats were allowed to explore the hole-board without holes for 10 min for habituation. After 24 h, a hole-board (39.8 × 39.8 × 5 cm) was inserted into the floor of the recording chamber. Each corner of the hole-board contained a hole. Then the rats were allowed to explore the hole-board for 10 min (first trial), and the number of holes nose-poke was counted. After 2 h, the same experimental context was maintained and the animals were allowed to re-explore the hole-board for 10 min (second trial) and the number of holes nose-poke was counted. To ensure that no odor cues were available, the recording chamber and hole-board were wiped with 20% alcohol after each trial. A reduction of head-dippings into the holes in the second trial indicates better recognition of novel context feature. For evaluating the dose and time dependent effects of iTBS on the HB performance, the first trial of the HB test started 30 or 80 min after real or sham-iTBS (*n* = 8 rats/group).

### 2.6. LFP recordings

The hippocampal LFP recordings were conducted 4 weeks after 6-OHDA injection. As previously described (Piilgaard and Lauritzen, 2009; Fordsmann et al., 2013; Li et al., 2014), Glass microelectrode (2–4 MΩ) filled with 1% pontamine sky blue in 0.5 M sodium acetate was placed in the left hippocampus (AP –5 mm, ML –3 mm, DV –3.2 mm; Paxinos and Watson, 1998). A screw placed on the occipital crest served as reference. The LFP signals were bandpass-filtered using a preamplifier (1–100 Hz) and digitized by the Spike 2 analysis system with sampling rate 100 Hz. After a 1 h stable baseline recording, the rats received sham-iTBS (*n* = 11 rats), 1 block-iTBS (*n* = 15 rats) or 3 block-iTBS (*n* = 11 rats), and then LFP was recorded for 2 h. At the end of the experiments, the LFP recording site was marked by iontophoretic ejection of pontamine sky blue through the microelectrode (−20 μA, 20 min).

### 2.7. Histology and immunohistochemistry

After completing different iTBS sessions described above, the rats were returned to their home cages for 30 or 80 min. Then, the rats were sacrificed and transcardially perfused with phosphate buffered saline (PBS) followed by 4% paraformaldehyde at 30 or 80 min post-stimulation. Brains were removed intact and post-fixed in the same fixative for 4 h, and immersed in a solution of 30% sucrose for 3 days. The brains were cut coronally into 35 μm thickness sections at the substantia nigra pars compacta (SNc), hippocampus and medial septum (MS). To determine the extent of dopaminergic neuron loss in the SNc and ventral tegmental area (VTA) in 6-OHDA-lesioned rats, tyrosine hydroxylase (TH) immunohistochemistry of the SNc and VTA was carried out. The sections of the SNc and VTA were washed three times in PBS for 10 min each and pre-incubated with 0.3% H_2_O_2_ for 30 min at room temperature. Then the sections were washed three times in PBS and incubated with 10% goat serum and 0.3% Triton X-100 in PBS for 1 h at room temperature. The sections were then incubated with rabbit anti-TH antibody (1:500; Abcam, Cambridge, UK) for 24 h at 4°C. After several washes, the sections were incubated with biotinylated goat anti-rabbit secondary antibody (1:4; ZSGB-Bio, China) for 1 h at 37°C. Sections were then washed again, treated with Horseradish Peroxidase conjugated streptavidin (1:4; ZSGB-Bio, China) for 1 h at 37°C, washed with PBS and incubated with 3, 3′-diaminobenzidine at room temperature for 10 s-1 min. Sections were mounted onto gelatin-coated slides, dehydrated, cleared in xylene and coverslipped. Only rats with a total loss of TH immunoreactivity in the left SNc were used to analyze behavioral data, electrophysiological recordings and immunofluorescence histochemistry.

To verify the location of the LFP recording site and identify basic neuronal structures of the hippocampus, the sections were stained with cresyl violet. To examine the density of c-Fos- and parvalbumin (PV)-positive neurons in the hippocampus, c-Fos and PV immunohistochemistry of the hippocampus were conducted in the six groups of rats (*n* = 6-7 rats/group). Further, we observed the density of PV-positive neurons in the MS (*n* = 4 rats/group), which is reciprocally connected with the hippocampus and thought to be the generator of theta rhythm. PV immunohistochemistry was applied in the MS and cresyl violet staining was carried out to identify basic neuronal structures of the MS. For c-Fos and PV immunohistochemistry, the serial sections of the hippocampus and MS were washed in PBS, and pre-incubated with 10% goat serum and 0.3% Triton X-100 in PBS for 1 h at room temperature. The sections were then incubated for 24 h at 4°C with mouse anti-c-Fos antibody (1:1,000; Abcam, Cambridge, UK) or mouse anti-PV antibody (1:500; Abcam, Cambridge, UK). After several washes, the sections were incubated with goat anti-mouse Alexa 555 (1:1,000; Millipore) secondary antibody for 4 h at room temperature. After staining, the sections were mounted in mounting medium containing DAPI (Beyotime Biotech, Shanghai, China). In order to verify the specificity of the staining, we did parallel control experiments where the primary antibodies for c-Fos and PV marker were omitted, respectively.

### 2.8. Data analysis and statistics

The power spectrum of LFP was computed with fast Fourier transform in a range of 0–50 Hz with 0.4 Hz of resolution (Villette et al., 2010). Theta power was calculated in 3–6 Hz. For quantitative analysis, peak theta frequency was considered to be the frequency corresponding to peak power in theta band, and normalized theta power was calculated with following formula: the power in the theta frequency band divided by the total power in the entire frequency spectrum (1–50 Hz) (Villette et al., 2010). Changes in peak theta frequency and normalized theta power were analyzed per 10 min before and after real or sham-iTBS using Spike 2 analysis system. The baseline value of peak theta frequency and normalized theta power were determined as the average value recorded in six 10-min epochs pre-stimulation, and then compared to the value in each 10-min epoch post-stimulation.

After TH staining, counting of the TH-immunoreactive (TH-ir) neurons in the SNc and VTA was carried out on representative three sections per rat as previously described (Wang et al., 2009). The number of c-Fos- and PV-labeled neurons for each rat brain was calculated from the average of the numbers from five serial sections corresponding to the major part of the dorsal hippocampus and MS. The digital images were captured from the sections at 10 × and 20 × magnification. The density of c-Fos- and PV-positive neurons (the number of c-Fos- and PV-positive neurons per mm2) was calculated in the left hippocampal and MS area.

Statistical analyses were performed with the Sigmaplot 14.0 version for Windows. The data of TH-ir neuron counts and the head-dippings in the HB test were compared using a paired Student’s *t*-test. The discrimination ratio in the OPR task and all the immunofluorescent data were analyzed by two-way analysis of variance (ANOVA) followed by *post hoc* comparisons based on Bonferroni’s test. One-way repeated ANOVA was used to analyze the hippocampal LFP data. Data were expressed as the mean ± SEM in the study. A two-tailed *P*-value of < 0.05 (α = 0.05) was considered significant.

## 3. Results

### 3.1. Dopaminergic neuron loss in 6-OHDA-lesioned rats

**FIGURE 2:**
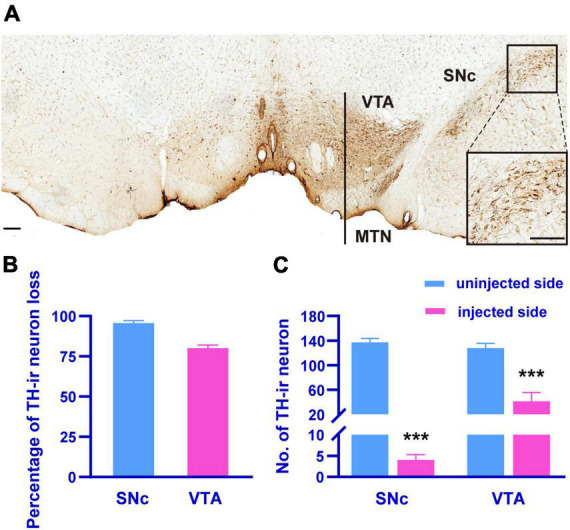
The TH-immunohistochemistry photomicrograph **(A)** showing the SNc and VTA dopaminergic neurons on the injected side (left) compared to uninjected side (right) in 6-OHDA-lesioned rats. The inset showing the zoomed image of selected region (black box). Histograms showing the percentage of TH-ir neuron loss in the SNc and VTA of the injected side compared to uninjected side in 6-OHDA-lesioned rats **(B)**, the differences in the number of TH-ir neuron in the SNc and VTA between the injected and uninjected side **(C)**. MTN, medial terminal nucleus of the accessory optic tract. Scale bar, *A* = 150 μm. ***p < 0.001 versus uninjected side.

The loss of dopaminergic neurons is a cardinal feature of PD. Therefore, we observed the loss of dopaminergic neurons by counting the number of TH-ir neurons in the SNc and VTA 4 weeks after the injection of 6-OHDA into the MFB (Figure 2A). The SNc on the lesioned side showed a total loss of TH-ir neurons compared to the unlesioned side (Figures 2B, C; paired *t*-test, *p* < 0.001), and the number of TH-ir neurons in the VTA on the lesioned side was reduced by 80.13 ± 6.05% of those on the unlesioned side (Figures 2B, C; paired *t*-test, *p* < 0.001).

### 3.2. The effects of different blocks of iTBS on hippocampus-dependent memory in the OPR task

**FIGURE 3:**
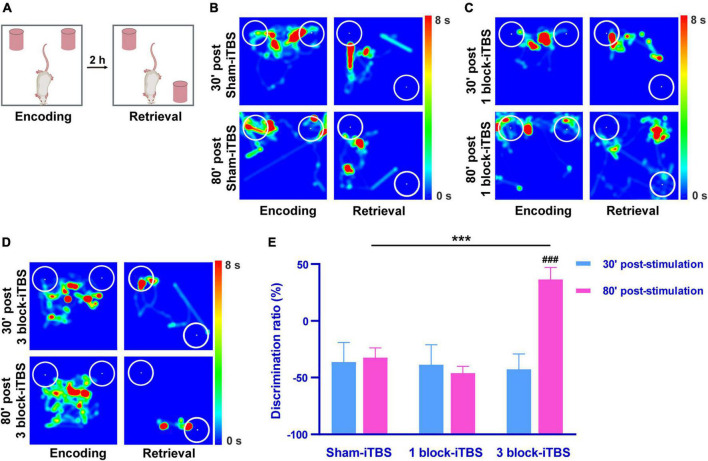
Schematic illustrations of the OPR task **(A)**. Heat map of the encoding phase and retrieval phase of the OPR task in rats receiving sham or real iTBS **(B–D)**. White circles: 2 cm area around the objects. Colors ranging from dark blue to dark red representing the exploration time of rats in the apparatus from 0 to 8 s. Histogram showing the discrimination ratio in the OPR task in rats receiving sham or real iTBS **(E)**. The discrimination ratio was significantly higher at 80 min post 3 block-iTBS group compared to 30 min post 3 block-iTBS or 80 min post sham-iTBS group, respectively **(E)**. There were no significant differences in the discrimination ratio among sham and 1 block-iTBS groups **(E)**. *n* = 7 rats/group. ***p < 0.001 versus 80 min post sham-iTBS group; ^###^*p* < 0.001 versus 30 min post 3 block-iTBS group.

The OPR task was carried out to assess hippocampus-dependent memory after application of sham or real iTBS. A higher discrimination ratio of the OPR task reflects greater memory retention for the stationary object place. Figure 3A shows schematic illustrations of the OPR task. As shown in Figures 3B–D, the rats tended to explore displaced object more time than the stationary one at 80 min after receiving 3 block-iTBS, but not sham-iTBS or 1 block-iTBS. A two-way ANOVA analysis (stimulation × time) showed a significant effect of stimulation on discrimination ratio for the treatment [*F*(2,41) = 5.07, *n* = 7 rats/group, *p* < 0.05], for the time [*F*(1,41) = 5.57, *n* = 7 rats/group, *p* < 0.05], and for their interaction [*F*(2,41) = 6.50, *n* = 7 rats/group, *p* < 0.01]. For between-group comparisons, subsequent *post hoc* analysis showed that 3 block-iTBS significantly increased the discrimination ratio at 80 min post-stimulation compared to sham-iTBS (Figure 3E; *p* < 0.001). 3 block-iTBS tended to decrease the discrimination ratio at 30 min post-stimulation compared to sham-iTBS, but this did not reach statistical significance (Figure 3E). 1 block-iTBS didn’t change the discrimination ratio at 30 or 80 min post-stimulation compared to sham-iTBS (Figure 3E). Within-group comparison between different time points showed a significant increase of the discrimination ratio at 80 min compared to 30 min after 3 block-iTBS (Figure 3E; *p* < 0.001). There were no significant differences in the discrimination ratio between 30 and 80 min post sham or 1 block-iTBS (Figure 3E).

### 3.3. The effects of different blocks of iTBS on hippocampus-dependent memory in the HB test

**FIGURE 4:**
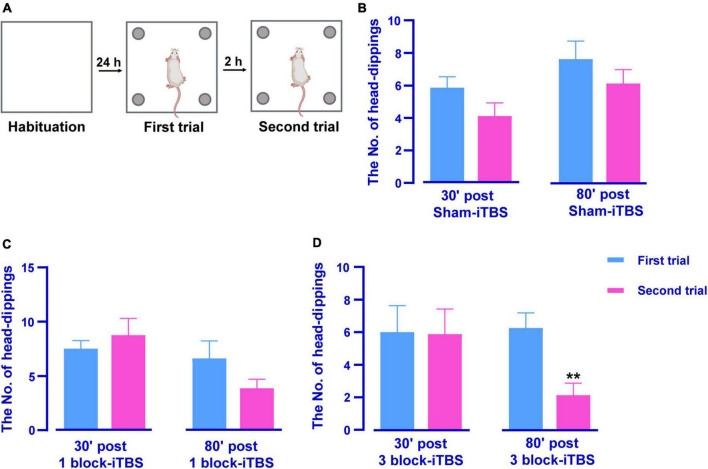
Schematic illustrations of the HB test **(A)**. Histogram showing the number of head-dippings in the HB test in rats receiving sham or real iTBS **(B–D)**. There were no significant differences in the number of head-dippings between first and second trail in sham and 1 block-iTBS groups **(B,C)**. The number of head-dippings was significantly lower in the second trial compared to the first trail in 80 min post 3 block-iTBS group **(D)**. *n* = 8 rats/group. **p < 0.01 versus first trail.

The HB test was also performed to evaluate hippocampus-dependent memory after sham or real iTBS. A reduction of head-dippings into the holes indicates better recognition of novel context feature. Figure 4A shows schematic illustrations of the HB test. Paired *t*-test showed 3 block-iTBS significantly decreased the number of head-dippings in the second trial compared to the first trail only at 80 min post-stimulation (Figure 4D; *n* = 8 rats/group, *p* < 0.01), not at 30 min (Figure 4D). Sham or 1 block-iTBS did not produce significant alterations in head-dipping behavior when the rats re-explored the hole-board at any time points (Figures 4B, C).

### 3.4. The effects of different blocks of iTBS on hippocampal theta rhythm

**FIGURE 5:**
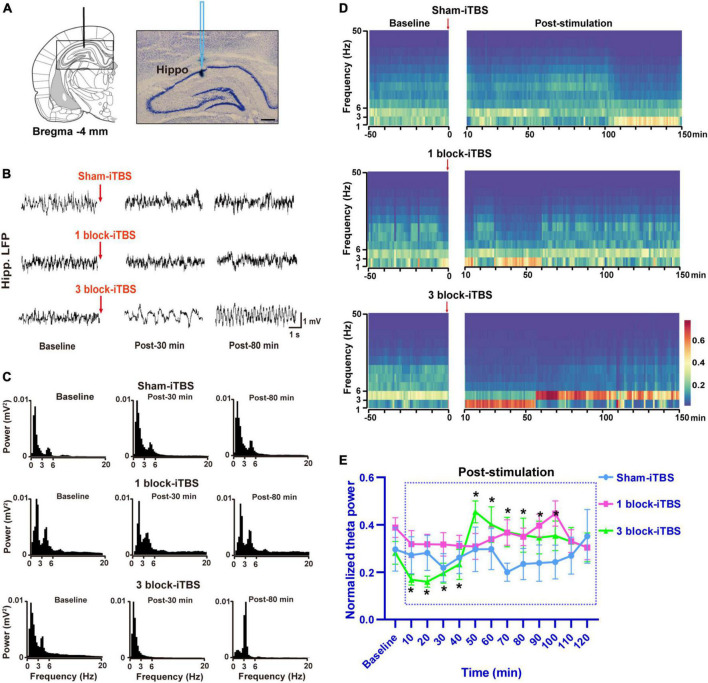
Schematic diagram and cresyl violet staining showing a representative slide of LFP recording from the dorsal hippocampus **(A)**. Example of raw LFP traces in the hippocampus during baseline, post-30 min, and post-80 min in sham-iTBS, 1 block-iTBS and 3 block-iTBS groups **(B)**. Example of power spectrum of LFP recorded in the hippocampus during baseline, post-30 min, and post-80 min in sham-iTBS, 1 block-iTBS and 3 block-iTBS groups **(C)**. Spectrograms of LFP recorded in the hippocampus before and after sham-iTBS, 1 block-iTBS or 3 block-iTBS **(D)**. Colors ranging from dark blue for low power to dark red for high power. Line graph showing the effects of sham-iTBS, 1 block-iTBS or 3 block-iTBS on normalized theta power **(E)**. Sham-iTBS or 1 block-iTBS didn’t change normalized theta power during a 2 h period post-stimulation compared to baseline **(E)**. 3 block-iTBS significantly decreased normalized theta power after 10 min, and this inhibitory effect lasted for 30 min, surprisingly, it significantly increased normalized theta power after 50 min, and this excitatory effect lasted for 50 min **(E)**. *n* = 11-15 rats/group. *p < 0.05 versus baseline normalized theta power. Scale bar, *A* = 400 μm.

**FIGURE 6:**
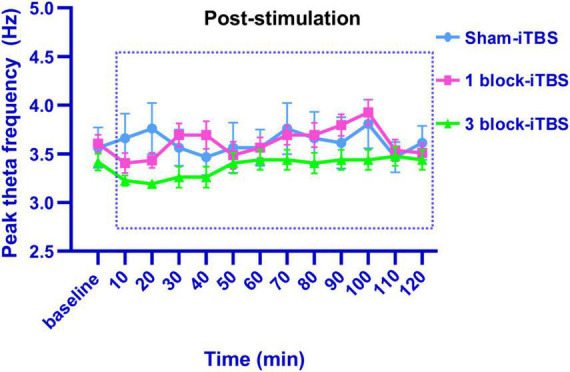
Line graph showing the effects of sham iTBS, 1 block-iTBS or 3 block-iTBS on peak theta frequency. Sham-iTBS or different blocks of iTBS didn’t affect peak theta frequency during a 2 h period post-stimulation compared to the pre-stimulation baseline.

In the present study, the LFP recording site used was verified to be within the hippocampus by iontophoretic ejection of pontamine sky blue (Figure 5A). Similar to the results in the behavioral tests, different blocks of iTBS also produced different effects on hippocampal theta rhythm. Sham-iTBS or 1 block-iTBS didn’t affect peak theta frequency or normalized theta power during a 2 h period post-stimulation compared to the pre-stimulation baseline (Figures 5B–E, 6). Surprisingly, 3 block-iTBS elicited a time-dependent effect on hippocampal theta rhythm [Figures 5B–E; *F*(12,141) = 4.93, *n* = 11 rats/group, *p* < 0.001]. It significantly decreased the normalized power of hippocampal theta rhythm after 10 min, and this inhibitory effect lasted for 30 min (Figure 5E; *p* < 0.05). Furthermore, it caused an increase in normalized theta power, which successively reached statistical significance at 50 min post-stimulation, and the excitatory effect lasted for 50 min (Figure 5E; *p* < 0.05). 3 block-iTBS didn’t change peak theta frequency during a 2 h post-stimulation compared to the baseline value (Figure 6).

### 3.5. The effects of different blocks of iTBS on protein expression

**FIGURE 7:**
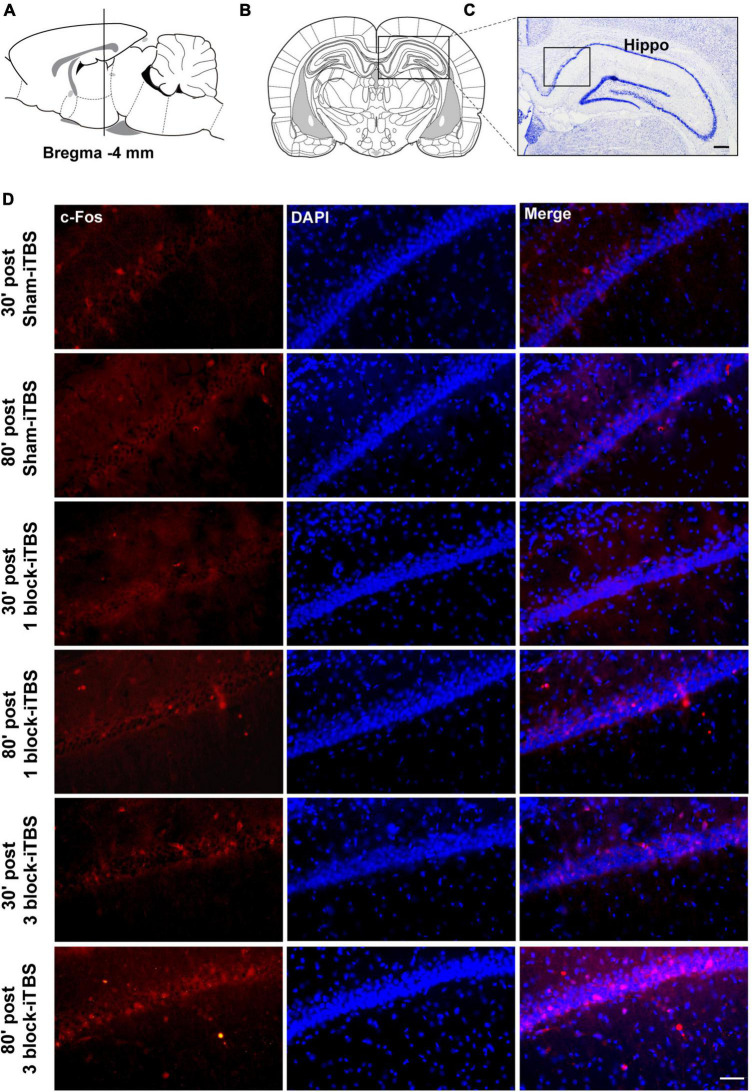
Schematic representation of sagittal **(A)** and coronal **(B)** sections comprising the hippocampus (adapted from Paxinos and Watson, 1998). Cresyl violet staining showing a representative slide of the hippocampus **(C)**. Representative immunofluorescence images of c-Fos (left, red), DAPI (middle, blue), and Merge (right, purple) in CA1 region of the hippocampus at 30 or 80 min post sham-iTBS, 1 block-iTBS or 3 block-iTBS **(D)**. Scale bar, *C* = 400 μm, *D* = 50 μm.

**FIGURE 8:**
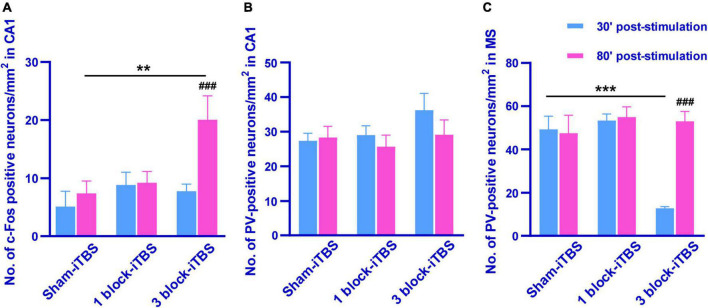
Histogram showing the density of c-Fos- and PV-positive neurons in the CA1 and MS **(A–C)**. The mean density of c-Fos-positive neurons in the CA1 was significantly higher in 80 min post 3 block-iTBS group than that in 30 min post 3 block-iTBS or 80 min post sham-iTBS group, respectively **(A)**. No significant difference in the mean density of PV-positive neurons in the CA1 was detected among sham-iTBS, 1 block-iTBS and 3 block-iTBS groups **(B)**. The mean density of PV-positive neurons in the MS was significantly lower in 30 min post 3 block-iTBS group than that in 80 min post 3 block-iTBS or 30 min post sham-iTBS group, respectively **(C)**. *n* = 4-7 rats/group. **p < 0.01, ***p < 0.001 versus sham-iTBS group, ^###^*p* < 0.001 versus 30 min post 3 block-iTBS group.

The density of c-Fos-positive neurons in the dorsal hippocampus was calculated after rats receiving sham or real iTBS. Representative sections of hippocampal immunofluorescent staining for c-Fos and DAPI are shown in Figure 7. A two-way ANOVA analysis (stimulation × time) showed a significant difference on the density of c-Fos-positive neurons in the dorsal hippocampus induced by iTBS for the treatment [*F*(2,38) = 2.80, *n* = 6-7 rats/group, *p* < 0.05], for the time [*F*(1,38) = 5.60, *n* = 6-7 rats/group, *p* < 0.05], but not for their interaction. Compared to sham-iTBS, 1 block-iTBS did not alter the density of c-Fos-positive neurons in the dorsal hippocampus regardless of whether 30 or 80 min post-stimulation (Figure 8A). Interestingly, changes in the density of c-Fos-positive neurons in the dorsal hippocampus induced by 3 block-iTBS showed a progressive increase over time compared to sham-iTBS. The mean density of c-Fos-positive neurons was still unchanged at 30 min post-stimulation compared to sham-iTBS (Figure 8A). At 80 min, the increase in the density of c-Fos-positive neurons induced by 3 block-iTBS was statistically significant (Figure 8A; *p* < 0.01).

**FIGURE 9:**
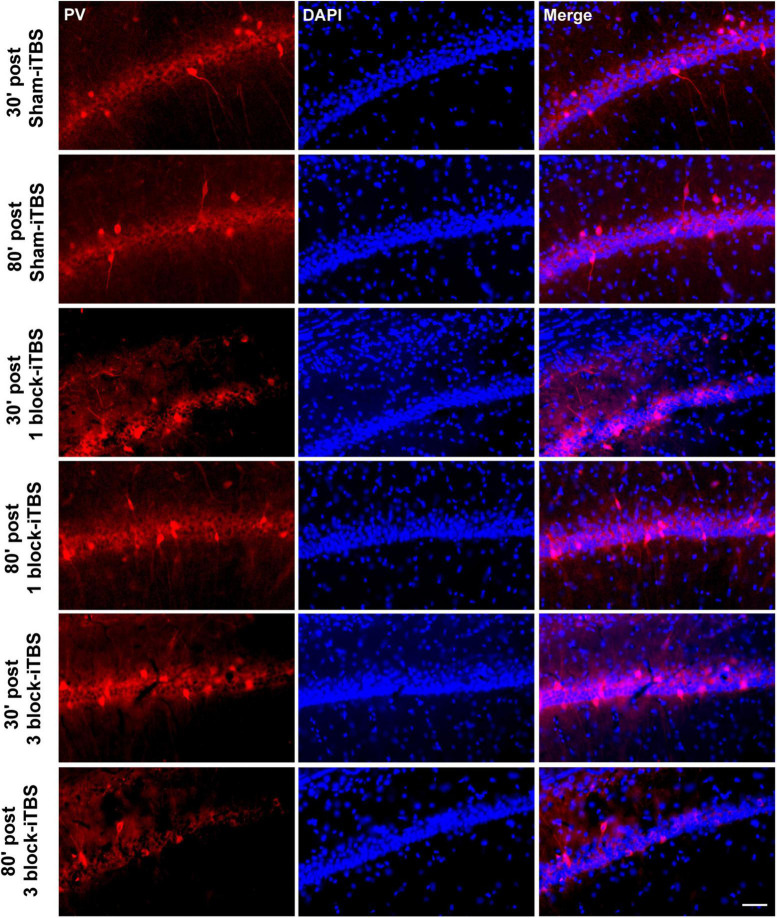
Representative immunofluorescence images of PV (left, red), DAPI (middle, blue), and Merge (right, purple) in the CA1 region of hippocampus at 30 or 80 min post sham iTBS, 1 block-iTBS or 3 block-iTBS. Scale bar = 50 μm.

**FIGURE 10:**
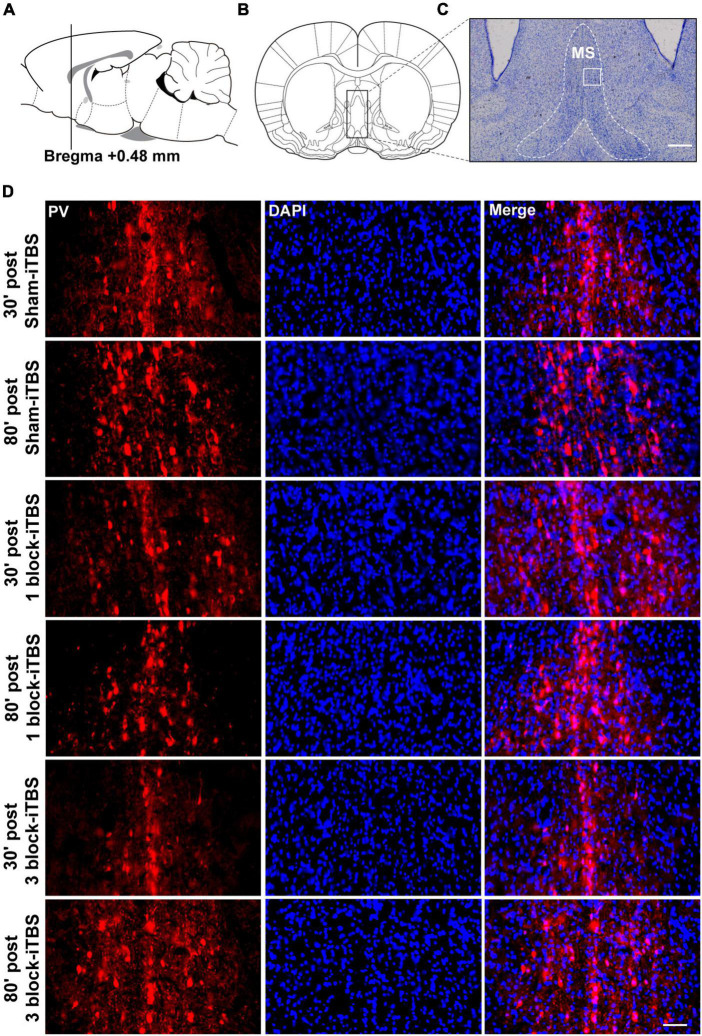
Schematic representation of sagittal **(A)** and coronal **(B)** sections comprising the MS (adapted from Paxinos and Watson, 1998). Cresyl violet staining showing a representative slide of the MS **(C)**. Representative immunofluorescence images of PV (left, red), DAPI (middle, blue), and Merge (right, purple) in the MS at 30 or 80 min post sham-iTBS, 1 block-iTBS or 3 block-iTBS **(D)**. Scale bar, *C* = 400 μm, *D* = 50 μm.

Furthermore, the density of PV-positive neurons in the dorsal hippocampus and MS were calculated after rats receiving sham or real iTBS. Representative sections of immunofluorescent staining for PV and DAPI in the dorsal hippocampus and MS are shown in Figures 9, 10. Unexpectedly, 3 block-iTBS did not alter the mean density of PV-positive neurons in the dorsal hippocampus (Figure 8B), but changed the mean density of those neurons in the MS (Figure 8C). A two-way ANOVA analysis (stimulation × time) showed a significant difference on the mean density of PV-positive neurons in the MS for the treatment [*F*(2,23) = 8.90, *n* = 4 rats/group, *p* < 0.01], for the time [*F*(1,23) = 9.95, *n* = 4 rats/group, *p* < 0.01] and for their interaction [*F*(2,23) = 10.09, *n* = 4 rats/group, *p* < 0.01]. *Post hoc* analysis showed that the mean density of PV-positive neurons in the MS was significantly lower at 30 min following 3 block-iTBS compared to sham-iTBS (Figure 8C; *p* < 0.001). However, 3 block-iTBS didn’t induce any change in the mean density of PV-positive neurons in the MS at 80 min post-stimulation compared to sham-iTBS (Figure 8C). Within-group comparison between different time points showed a significant increase in the mean density of PV-positive neurons in the MS at 80 min compared to 30 min after 3 block-iTBS (Figure 8C; *p* < 0.001). 1 block-iTBS did not change the density of PV-positive neurons in the dorsal hippocampus or MS when compared to sham-iTBS (Figure 8C).

## 4. Discussion

The main results of this study are as follows: (i) Compared to sham-iTBS, 1 block-iTBS didn’t change the discrimination ratio in the OPR task and the number of head-dippings when re-exposure to the HB test, however, 3 block-iTBS significantly increased the discrimination ratio in the OPR task, and decreased the number of head-dippings when re-exposure to the HB test at 80 min post-stimulation but not 30 min; (ii) Sham-iTBS and 1 block-iTBS had no effects on peak theta frequency and normalized theta power. Interestingly, 3 block-iTBS significantly decreased the normalized theta power at 10 min post-stimulation, and this inhibitory effect lasted for 30 min, subsequently, it caused a significant increase of theta power at 50 min post-stimulation, and the excitatory effect lasted for 50 min; and (iii) 1 block-iTBS didn’t alter the density of c-Fos- or PV-positive neurons in the hippocampus and MS compared to sham-iTBS. 3 block-iTBS significantly increased the density of c-Fos-positive neurons in the hippocampus at 80 min post-stimulation, and decreased the density of PV-positive neurons in the MS at 30 min post-stimulation compared to sham-iTBS. These results suggest that 3 block-iTBS but not 1 block-iTBS enhanced memory performances measured by the OPR and HB test. Further, the effects of iTBS were time-dependent. These findings might be explained by the following reasons.

First, there is a large body of ablation or electrophysiological evidence about the fundamental role of the hippocampus in OPR memory and HB performances (Manns and Eichenbaum, 2009; Barker and Warburton, 2011; Costa et al., 2012; Liu et al., 2019). Bilateral hippocampal lesions significantly impaired OPR memory (Barker and Warburton, 2011). A previous study revealed that the pattern of activity among many simultaneously recorded hippocampal neurons reflected object location information (Manns and Eichenbaum, 2009). In hemiparkinsonian rats, a study showed that impaired performance in the HB test was attributed to altered hippocampal long-term potentiation (Costa et al., 2012), and our previous study reported that blockade of hippocampal serotonin6 receptor enhanced HB performance (Liu et al., 2019). These findings suggest that the hippocampus participates in the process of memory performances assessed by the OPR and HB test. Additionally, emerging evidence from experimental, clinical imaging and pathological research indicates a relationship between cognitive decline and hippocampal structural/functional abnormalities in PD (Calabresi et al., 2013; Kouli et al., 2020; Becker et al., 2021; Jiang et al., 2021). Hippocampal atrophy and Lewy body pathology in the hippocampus have been shown to be associated with cognitive dysfunction in PD (Camicioli et al., 2003; Costa et al., 2012; Pereira et al., 2013). Further, our previous study found that decreased hippocampal theta power was an important cause of memory impairment in parkinsonian rats (Li et al., 2015). These studies suggest that the hippocampus involves in cognitive impairment in PD, and specific regulation of hippocampal activity may affect PD-related cognitive behaviors assessed by the OPR and HB test. In the present study, we found that 3 block-iTBS significantly increased the density of c-Fos-positive neurons in the hippocampus at 80 min post-stimulation. c-Fos, the product of the immediate expression gene c-fos, has been widely used as a marker for neuronal activation (Nikolaev et al., 1992). Thus, an increase in c-Fos expression in the hippocampus induced by 3 block-iTBS means that 3 block-iTBS can activate hippocampal neurons, thereby leading to the enhancement of hippocampus-dependent memory in PD at 80 min post-stimulation. Besides, the recognition of familiar spatial rearrangements and novelty discrimination in the OPR task were found to be accompanied with an increase of c-Fos level in the hippocampus (Mendez et al., 2015; Wang C. et al., 2021), suggesting that increased hippocampal c-Fos level may be causally linked to the enhancement of OPR memory. This may also help explain why 3 block-iTBS improved OPR memory performance in PD at 80 min post-stimulation. In addition, we found that 1 block-iTBS didn’t change the density of hippocampal c-Fos-positive neurons, which might account for unaltered memory behaviors after 1 block-iTBS.

Second, hippocampal theta rhythm is considered to be a physiological encoding frequency in memory encoding and reinstatement (Buzsáki, 2002). Sato and Yamaguchi (2005) found that hippocampal theta phase encoding was involved in the storage of object-place associations. Further, Boyce et al. (2016) showed that impaired novel OPR memory was associated with the interruption of hippocampal theta rhythm by using optogenetic method. More recently, novelty discrimination in the recognition memory task was found to be accompanied with higher theta activity in the hippocampus (Wang C. et al., 2021). These findings provide a solid evidence base for the positive role of theta rhythm in hippocampus-dependent memory. Our data showed that 3 block-iTBS increased normalized theta power at 50 min post-stimulation, and the excitatory effect lasted for 50 min, which supports our results showing that 3 block-iTBS enhanced hippocampus-dependent memory at 80 min post-stimulation. Surprisingly, we found that 3 block-iTBS first decreased and then increased normalized theta power during a period of 2 h following stimulation. This leads to an intriguing question: why did 3 block-iTBS induce bidirectional effect on theta power? Apart from the hippocampus, we also observed protein changes in the MS which plays a critical role in the production of hippocampal theta rhythm (Yoder and Pang, 2005; Müller and Remy, 2018). Activation of the MS induced hippocampal theta power (Goutagny et al., 2008; Gangadharan et al., 2016; Wang Y. et al., 2021), and lesions or inactivation of the MS disrupted hippocampal theta rhythm (Rawlins and Olton, 1982; Givens and Olton, 1990; Roland et al., 2014). In this case, the preliminary inhibition of hippocampal theta power induced by 3 block-iTBS could be explained on the basis of reduced PV expression in the MS. The calcium-binding protein PV, specific to fast-spiking interneurons (Markram et al., 2004), signals the metabolic and synaptic activity of neurons (Patz et al., 2004) and a loss of PV expression means a reduced activity state of interneurons (Bender et al., 2000; Tropea et al., 2006; Schwaller, 2009). Moreover, the PV-positive neurons are a main group of GABAergic neurons in the MS, and serve as “pacemakers” of hippocampal theta rhythm (Freund and Antal, 1988; Borhegyi et al., 2004). Therefore, changes in MS PV-positive neurons can alter theta rhythm. In the present study, we note that 3 block-iTBS decreased the density of MS PV-positive neurons at 30 min post-stimulation and restored it at 80 min post-stimulation, which may result in a transient inhibition of hippocampal theta rhythm. We also observed that 3 block-iTBS increased theta power following the inhibition period. What might account for this excitatory effect? It has been reported that theta generation involves the disinhibition of hippocampal pyramidal neurons (Colgin, 2016). In the present study, 3 block-iTBS induced an increase in the density of hippocampal c-Fos-positive neurons at 80 min post-stimulation, reflecting the activation of hippocampal neurons, which may be responsible for the enhancement of theta power.

Third, dopamine (DA) neurotransmission in the dorsal hippocampus is involved in a range of functions from learning to memory (O’Carroll et al., 2006; Rossato et al., 2009; Bethus et al., 2010). Previous study showed that a simultaneous release of DA in the hippocampus was observed during object location memory, while depletion of catecholaminergic terminals in the hippocampus by 6-OHDA impaired object location memory (Moreno-Castilla et al., 2017). In parkinsonian animal, DA precursor levodopa was able to ameliorate the cognitive deficit measured by the HB test (Costa et al., 2012). These findings indicate that DA can promote hippocampus-dependent memory. This begs the following question, can iTBS affect memory *via* regulating DA level? An iTBS study in parkinsonian rats validated this hypothesis. It was shown that acute iTBS altered DA level in the striatum in a temporal-specific manner, with a positive peak around 80 min post-stimulation (Cacace et al., 2017), which may explain the time-dependent effect induced by 3 block-iTBS.

In this study, we aim to elucidate the underlying mechanism involved in the effect of iTBS and attempt to uncover this mechanism through protein changes in c-Fos and PV. To some extent, PV could reflect activity state of interneurons, however, it might be better to further analyze changes in the co-expression of PV and c-Fos in neurons induced by iTBS.

## 5. Conclusion

Our findings indicate that multiple blocks of iTBS elicit dose and time-dependent effects on hippocampus-dependent memory in PD. 1 block-iTBS has no effect on hippocampus-dependent memory in PD. 3 block-iTBS couldn’t affect hippocampus-dependent memory at 30 min post-stimulation but enhances it at 80 min, which indicates that 3 block-iTBS causes a distinct profile at different time-point. These dose and time-dependent effects are likely due to changes in c-Fos expression, normalized theta power and DA level in the hippocampus.

## Data availability statement

The raw data supporting the conclusions of this article will be made available by the authors, without undue reservation.

## Ethics statement

The animal study was reviewed and approved by the Ethics Committee for Animal Experimentation of Xi’an Jiaotong University.

## Author contributions

LL and JL conceived the study. LL and QZ acquired the funding and designed the experiments. LL and YW conducted the experiments and performed statistical analysis and edited the manuscript. YH, ZW, and LW provided resources. ZW, XW, and YB were involved in software writing. LL wrote the original draft of the manuscript after a fruitful discussion with YH, ZW, and LW. QZ and JL reviewed the manuscript. All authors have read and approved the submitted manuscript.
